# Application of Intelligent Detection of Neural Signal in Depth Evaluation of Obstetrics and Gynecology Anesthesia

**DOI:** 10.1155/2022/6027965

**Published:** 2022-03-23

**Authors:** Xiushuang Zhang, Mingjun Xu, Xiaoguang Li, Xiuling Cao, Xiangming Che

**Affiliations:** Beijing Obstetrics and Gynecology Hospital, Capital Medical University, Beijing 100026, China

## Abstract

In order to evaluate the application of EEG intelligent detection in gynecological anesthesia depth, the application of ANGEL-6000 EEG depth monitor in laparoscopic gynecological anesthesia was proposed. This method was applied to 60 patients who underwent elective laparoscopic gynecological surgery in our hospital from February to August 2016. Inclusion criteria were ASA i ∼ ii; the average age was (37.8 ± 6.6) years from 20 to 50 years old; the average body weight was (51.53 ± 3.87) kg; conscious and no communication barriers; and patients without instrument ventilation. The patients were divided into observation group and control group according to the random number table method, with 30 patients in each group. The two groups were anesthetized with the same anesthetic drugs, and their consciousness index was monitored. IoC values were recorded before induction of anesthesia (T0), 5 min after intubation (T1), 5 min after operation (T2), intraoperative exploration (T3), at the end of operation (T4), 1 min before extubation (T5), and 5 min after extubation (T6). The dosage of anesthetic drugs, operation time, extubation time, and operation time of the two groups were statistically analyzed. Compared with the operation time of patients in the two groups, the extubation time, awake time, and time out of the operating room of patients in the control group were longer than the observation group. The IoC values of patients in the control group at T0 and T6 time points were lower than those in the observation group at each time point from T1 to T5. Comparison of perioperative dose of remifentanil and atracurium between the two groups was performed. The control group used more propofol dose in perioperative period. The application of neuroelectric signal in laparoscopic gynecological surgery to detect changes in perioperative IoC value can well reflect the level of consciousness of patients and reflect the effect of perioperative stimulation at different time points on the EEG of patients in real time.

## 1. Introduction

Anesthesia is an important part of modern clinical medicine. The monitoring of the depth of anesthesia (DOA) is helpful to control the anesthetic dose and achieve the best anesthetic effect with the least amount of anesthetic drugs. It cannot only prevent the danger caused by the overdose of anesthetic drugs and shorten the recovery process but also avoid intraoperative awareness. Appropriate depth of anesthesia is the key to ensuring the safety of patients and creating good operating conditions for surgery [1]. Anesthesia was caused by reversible inhibition and excitement of the central nervous system so as to achieve the aim of unconsciousness and the pain, and cerebral cortex pyramidal top dendritic cells which produce electricity is the sum of the dendritic potentials and postsynaptic potential and can directly reflect the activities of the central nervous system, so the electrical testing analysis is the best way to determine the depth of anesthesia. The application of EEG in anesthesia monitoring marks the beginning of the application of EEG in anesthesia [2]. After the 1960s, with the development of computers and signal processing technology, EEG recording and analysis technology has been continuously improved. In the field of clinical anesthesia, both primitive EEG, quantitative EEG, and evoked potential monitoring have different applications [3]. Evoked potentials used in depth detection of anesthesia mainly include auditory evoked potentials and somatosensory evoked potentials, and EEG analysis methods mainly include EEG dual frequency index, EEG complexity analysis, and EEG entropy analysis. Auditory evoked potentials (AEPs) are the potential activities recorded by the scalp and conducted through auditory pathways after sound stimulation. In the awake state, the differences among individuals are very small [4]. Comprehensive studies have shown that auditory evoked potentials can be used as a sensitive indicator of cerebral cortex information processing and cognitive function during general anesthesia. Since both intraoperative awareness and inadequate depth of anesthesia can be recorded, auditory evoked potentials can be used to monitor depth of anesthesia. Auditory evoked potentials from the cochlea and various auditory centers within 10 ms with incubation period were recorded from the cranial top by means of short sound stimulation to the human ear and cumulative average technique [5]. Since anesthesia can be produced by different mechanisms, it is more reliable to use multidimensional parameters to represent the changes of brain state during anesthesia than a single parameter [6]. Using a variety of methods and multiparameter joint monitoring of anesthesia depth may be the way to achieve accurate monitoring of anesthesia depth [7].

To answer this research question, Shang et al. evaluated and compared the Narcotrend, BIS, and traditional EEG monitoring during induction, maintenance, and recovery of anesthesia by propofol combined with remifentanil, confirmed that Narcotrend and BIS can accurately distinguish each anesthesia stage (awake, no response, no eye-opening response, clinical anesthesia state, first response, and extubation during anesthesia recovery), and predicted the general effect rate >0.95. There were significant changes in hemodynamics after the initiation of remifentanil infusion. Compared with traditional EEG and hemodynamic parameter monitoring methods, Narcotrend and BIS of modern EEG monitoring system can reliably evaluate and distinguish each anesthesia stage from induction of anesthesia to extubation of anesthesia recovery but cannot reflect the level of analgesia in each anesthesia stage [8]. Guerrero-Hernández et al. conducted a prospective, random, and double-blind comparative study of Narcotrend index or BIS with traditional monitoring methods in desflurane-combined remifentanil anesthesia and confirmed that the Narcotrend group and BIS group have the same effect, and the dosage of desflurane is significantly reduced compared with the traditional group. During the recovery stage of desflurane combined with remifentanil, the Narcotrend grade is correlated with the desflurane end-expiratory concentration, and with the typical change of the trend from C/D/E to A/B/C, the desflurane end-expiratory concentration gradually decreases [9]. Nakazato et al. confirmed that under desflurane anesthesia in different age groups, EC50 in children was significantly higher than that in young people and adults. Narcotrend is a better predictor of anesthetic depth than cEEG, MAP, and HR. In children's sevoflurane anesthesia, a randomized control study was conducted on the correlation between the Narcotrend index and end-expiration concentration of sevoflurane and hemodynamic parameters, and it was confirmed that the Narcotrend index was more related to the unstable end-expiration concentration of sevoflurane in children [10]. At present, bispectrum index and auditory evoked potential (AAI) index are the most widely used in clinical practice. Auditory evoked potentials can quickly reflect the loss and recovery of consciousness of patients, predict motor responses and intraoperative awareness, and are not affected by neuromuscular blockers but are not suitable for patients with auditory pathway injury. Although EEG bispectral index can reflect the depth of anesthesia sensitively, it has the shortcoming of reflecting different anesthesia drugs and methods, so it cannot be a perfect technique for clinical anesthesia monitoring independently. On the basis of the current research, this paper proposed to explore the application of ANGEL-6000 EEG depth monitor in laparoscopic gynecological surgery anesthesia. This method was applied to 60 patients who were selected for laparoscopic gynecological surgery in our hospital from February to August 2016. Inclusion criteria were as follows: ASA i ∼ ii; the average age was (37.8 ± 6.6) years from 20 to 50 years old; the average body weight was (51.53 ± 3.87) kg; conscious, no communication barriers; and patients with noninstrument ventilation [11]. According to random number table method, they were divided into the observation group and control group, 30 cases in each group. The two groups were anesthetized with the same anesthetic drugs, and their consciousness index was monitored. IoC values were recorded before induction of anesthesia (T0), 5 min after intubation (T1), 5 min after operation (T2), intraoperative exploration (T3), at the end of operation (T4), 1 min before extubation (T5), and 5 min after extubation (T6). The dosage of anesthetic drugs, operation time, extubation time, and operation time of the two groups were statistically analyzed. Compared with the operation time of patients in the two groups, the extubation time, awake time, and time out of the operating room of patients in the control group were longer than the observation group. The IoC values of patients in the control group at T0 and T6 time points were lower than the observation group at each time point from T1 to T5, the difference was statistically significant (*p* < 0.001). There was no statistical significance in the perioperative dose of remifentanil and atracurium between 2 groups (*p* > 0.05). The control group used more propofol dose in the perioperative period, and the difference was statistically significant (*p* < 0.001) [12]. The application of neural electrical signals in laparoscopic gynecological surgery to intelligently detect changes in perioperative IoC values of patients can well reflect the level of consciousness of patients and reflect in real time the effects of perioperative stimulation at different time points on patients' EEG [13].

## 2. Data and Methods

### 2.1. General Information

**Figure 1:**
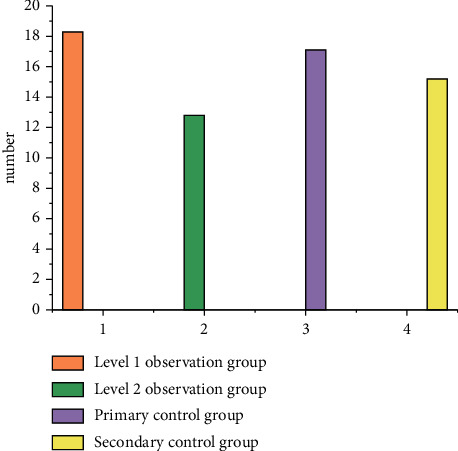
Data of the experimental group and control group.

Sixty patients undergoing elective laparoscopic gynecological surgery in our hospital from February to August 2016 were selected, and the inclusion criteria were as follows: ASA i ∼ ii; the average age was (37.8 ± 6.6) years from 20 to 50 years old; the average body weight was (51.53 ± 3.87) kg; conscious, no communication barriers; patients without instrument ventilation; discharge of coagulation disorders; and patients with serious heart and lung diseases, liver and kidney dysfunction, neurological diseases, history of drug abuse, alcoholism, and poor compliance. According to the random number table method, they were divided into observation group and control group, 30 cases in each group. There were 18 ASA i patients and 12 ASA ii patients in the observation group, with an average age of (36.6 ± 5.4) years and an average body weight of (50.84 ± 4.13) kg. In the control group, there were 16 ASA grade i patients and 14 ASA grade ii patients, with an average age of (37.5 ± 5.4) years and an average body weight of (51.84 ± 4.86) kg, as shown in Figure 1. There was no significant difference in age, body weight, and other general information between the two groups (*p* > 0.05), indicating comparability.

### 2.2. Methods

#### 2.2.1. Preoperative Preparation and Monitoring

The patient did not receive preoperative drugs. After entering the operating room, the peripheral vein was routinely opened and the patient was given sodium Ringer's lactate solution 10 mL/kg. At the same time, the patient's electrocardiogram (ECG), heart rate (HR), finger pulse oxygen saturation (SpO_2_), and noninvasive blood pressure (SDP/DBP) were monitored by a multifunctional monitor, and vital sign data were collected every 3 minutes. Angel-6000 EEG depth monitor was used to monitor the anesthesia depth of patients, and the corresponding IoC value was collected. The skin on the forehead and zygomatic bone of patients in the two groups was cleaned, and the IoC monitoring electrode was affixed to the middle of the forehead, the right side of the forehead, and the zygomatic bone to monitor changes in IoC values of patients [14].

#### 2.2.2. Methods of Anesthesia

Propofol (AstraZeneca UK Limited, h20130535), fentanyl (AstraZeneca UK Limited, h20030197), and atracurium (AstraZeneca UK Limited, h20060869) were given to the two groups to maintain and monitor the IOC value of the patients. In the control group, anesthesia was induced and maintained according to the clinical experience of anesthesiologists and patients' vital signs, but the use of narcotics was not guided by IoC feedback during the process. In the observation group, the depth of anesthesia was monitored by angel-6000 EEG anesthesia depth monitor to monitor the IoC value, and the dosage of narcotic drugs was adjusted through its IoC feedback to maintain the IoC value between 40 and 60. Specific plan: in control patients vein pump injection of propofol was performed, dosage was according to the anesthesiologist clinical experience and the patient's blood pressure and heart rate change, and then fentanyl mu 2 g/kg and atracurium 1 mg/kg was administered intravenously, after complete perfect muscle relaxation of patients. Auxiliary downward endotracheal intubation in laryngeal mirror was performed, the endotracheal tube was confirmed to be in place to be connected with the breathing machine for mechanical control of ventilation, and the continuous end-expiratory pCO_2_ was kept between 30 and 35 mmHg. After induction of anesthesia, infusion of propofol, remifentanil, and atracurium micropump was continued until the end of surgery. Remifentanil and atracurium were injected at a constant rate of 10 *μg*/(kg·h) and 0.5 mg/(kg·h), respectively, while propofol was initially injected at a rate of 5 mg/(kg·h). Then, the anesthesiologist decided the infusion rate of propofol based on clinical experience and changes in patients' blood pressure and heart rate. 5–10 mg ephedrine intravenous injection was given to patients with hypotension during surgery. If the patient's heart rate was less than 50 beats/min, 0.5 mg atropine was given intravenously. The induction dose and maintenance dose of propofol in the observation group were adjusted according to the IoC value monitored by angel-6000 EEG depth monitor, and the IoC value was maintained between 40 and 60. The dosage and infusion rate of remifentanil and atracurium were the same as those in the control group [15]. Atracurium infusion was stopped when pneumoperitoneum was relieved, and tramadol 100 mg was given intravenously. All narcotic drugs were stopped at the end of surgery. After surgery, patients were given the Steward score. The tracheal catheter was removed after the Steward score was ≥4. No obvious abnormality was observed for 5 min after tracheal tube removal, and the patient was sent back to the ward for observation and subsequent treatment [16].

### 2.3. Observation Indicators

The dosage of narcotic drugs, operation time, extubation time, waking time, and time to leave the operating room were observed and recorded in the two groups. Meanwhile, IoC values at different time points during perioperative anesthesia induction (T0), 5 min after intubation (T1), 5 min after operation (T2), intraoperative exploration (T3), end of operation (T4), 1 min before extubation (T5), and 5 min after extubation (T6) were recorded.

## 3. Results and Analysis

### 3.1. Comparison of Operation Time, Extubation Time, Awake Time, and Time to Leave the Operating Room between the Two Groups

There was no significant difference in operation time between 2 groups (*p* > 0.05). The extubation time, awake time, and time to leave the operating room in the observation group were shorter than those in the control group, and the differences were statistically significant (*p* < 0.05). (see Table 1).

### 3.2. Comparative Analysis of IoC Values between the Two Groups at Different Time Points

At T0 and T6 time points, there was no significant difference in IoC values between the two groups (*p* > 0.05). IoC values of patients in the observation group were higher than those in the control group at each time point from T1 to T5, and the difference was statistically significant (*p* < 0.001) as shown in Table 2.

Comparison of perioperative anesthetic dose between 2 groups: there was no statistical significance in the perioperative dose of remifentanil and atracurium between 2 groups (*p* > 0.05). The perioperative dose of propofol in the observation group was less than that in the control group, and the difference was statistically significant (*p* < 0.001) (see Table 3).

Gynecological laparoscopic surgery can avoid the traditional operation of laparotomy, reduce the injury of the patient's body, and reduce the degree of postoperative pain, at the same time, reduce the incidence of postoperative complications, shorten the length of hospital stay, and help patients recover quickly, therefore, welcomed by doctors and patients. During laparoscopic surgery, pneumoperitoneum should be established to maintain the pneumoperitoneum pressure between 10 and 13 mmHg to avoid unbearable discomfort during epidural or spinal anesthesia, while general anesthesia or combined spinal anesthesia are common anesthesia methods in clinical gynecology [17]. Gynecological laparoscopic surgery anesthesia, if the depth of anesthesia is shallow, can lead to awareness of the patient, knowing of the operation process, and lead to postoperative psychological disorders and mental dysfunction; when anesthetic depth is deeper, it will prolong the patient's waking time, and in severe cases, it will damage the patient's neurological function. In this study, propofol, remifentanil, and atracurium were used as anesthesia induction and maintenance. Propofol is a commonly used anesthetic in clinical practice, which can sedate patients, and the degree of sedation is related to the dosage used. Previous studies have shown that with the increase of the blood concentration of propofol in patients, the consciousness of patients gradually decreased and disappeared. Remifentanil is an ultra-short-acting and potent opioid analgesic drug with a short half-life and strong analgesic effect [18]. Atracurium is a medium-effect nondepolarizing muscle relaxant. Combined use of the three drugs can provide analgesia, sedation, and muscle relaxation, and the effect is precise and controllable. The depth of anesthesia is monitored by the electroencephalogram (EEG) of patients monitored by angel-6000 electroencephalograph, and the high-frequency converted energy during anesthesia is calculated by the special EEG power spectrum *α* and *β* differences in ratios. At the same time, the instantaneous burst of each cycle and the “static” and “mild” general anesthesia depth levels in the quantitative stage were calculated in real time, and the anesthesia depth index was calculated with four parameters. IoC results used 0–100 scale to indicate the degree of fuzzy consciousness of patients, and 40–60 scale was the most appropriate level of anesthesia for surgery. Studies have shown that IoC value decreases with the increase of blood propofol concentration [19]. The results of this study showed that the extubation time, waking time, and time out of the operating room of patients in the observation group were shorter than those in the control group, indicating that the Angel-6000 EEG depth monitor can provide clinical anesthesiologists with objective and real-time consciousness index, enabling anesthesiologists to adjust the anesthesia depth according to its IoC value. Meeting the operation requirements of anesthetic sedative degree and achieving the purpose of patients round tube drawing as soon as possible also can effectively control narcotics use dose, by monitoring the IoC values, assess the depth of anesthesia, and to achieve reasonable after anesthesia depth decrease narcotic drugs continue to pump injection, avoid judgment, based on the experience of clinical anesthesia depth caused by narcotic drugs given excessive, and result in delayed awakening. The results of this study suggested that IoC values of patients in the observation group were higher than those in the control group at each time point from T1 to T5, and the dosage of propofol in the observation group was lower, suggesting that the dosage of propofol in the control group was high, resulting in the depth of anesthesia in patients. IoC value is used as an objective index of anesthesia depth to provide feedback and guidance for anesthesiologists in the use of anesthetic dose, which can also improve the anesthetic effect of patients and shorten the time of anesthesia awakening, extubation, and leaving the operating room [20].

## 4. Conclusion

In this paper, the application of Angel-6000 EEG depth monitor in laparoscopic gynecological anesthesia was proposed. This method was applied to 60 patients who were selected for laparoscopic gynecological surgery in our hospital from February to August 2016. Inclusion criteria were as follows: ASA I∼II; the average age was (37.8 ± 6.6) years from 20 to 50 years old; the average body weight was (51.53 ± 3.87) kg; conscious, no communication barriers; and patients without instrument ventilation. According to the random number table method, they were divided into the observation group and control group, 30 cases in each group. The two groups were anesthetized with the same anesthetic drugs, and their consciousness index was monitored. IoC values were recorded before induction of anesthesia (T0), 5 min after intubation (T1), 5 min after operation (T2), intraoperative exploration (T3), at the end of operation (T4), 1 min before extubation (T5), and 5 min after extubation (T6). The dosage of anesthetic drugs, operation time, extubation time, and operation time of the two groups were statistically analyzed. Compared with the operation time of patients in the two groups, the extubation time, awake time, and time out of the operating room of patients in the control group were longer than those in the observation group. The IoC values of patients in the control group at T0 and T6 time points were lower than those in the observation group at each time point from T1 to T5, the difference was statistically significant (*p* < 0.001). There was no statistical significance in the perioperative dose of remifentanil and atracurium between 2 groups (*p* > 0.05). The control group used more propofol dose in the perioperative period, and the difference was statistically significant (*p* < 0.001). The application of the neuroelectric signal in laparoscopic gynecological surgery to detect changes in the perioperative IoC value can well reflect the level of consciousness of patients and reflect the effect of perioperative stimulation at different time points on the EEG of patients in real time. At present, bispectrum index and auditory evoked potential (AAI) index are the most widely used methods for monitoring anesthesia depth. Each method has its advantages and limitations. Auditory evoked potentials can quickly reflect the loss and recovery of consciousness of patients, predict motor responses and intraoperative awareness, and are not affected by neuromuscular blockers but are not suitable for patients with auditory pathway injury. Although EEG bispectral index can reflect the depth of anesthesia sensitively, it has the shortcoming of reflecting different anesthesia drugs and methods, so it cannot be a perfect technique for clinical anesthesia monitoring independently. Both complexity and entropy are nonlinear dynamic analysis methods, which can extract information about changes in anesthesia depth and have broad application prospects. However, they have not been widely applied and promoted in clinical practice, and their advantages and influencing factors need further study.

## Figures and Tables

**Table 1:** Comparison of operation time, extubation time, awake time, and time to leave the operating room between the two groups.

Group	Operation time	Extubation time	Waking hours	Time to leave the operating room
Observation group	70.43	7.79	4.59	14.25
Control group	71.56	12.99	9.89	17.59
t	−0.244	−0.4508	−5.921	−3.458

**Table 2:** Comparative analysis of IoC values between the two groups at different time points.

Group	T0	T1	T2	T3	T4	T5	T6
Observation group	96.99	56.48	57.34	58.77	58.56	89.66	96.01
Control group	97.58	42.53	45.39	43.62	51.452	72.984	95.23
t	−1.629	14.356	8.59	15.541	4.084	7.752	1.865

**Table 3:** Comparative analysis of perioperative anesthetic dose between the two groups.

Grouping	Propofol	Fentanyl	Atracurium
Observation group	257.63	684.76	80.64
Control group	503.48	678.59	79.12
t	−9.535	0.125	0.473

## Data Availability

The data used to support the findings of this study are available from the corresponding author upon request.
